# Multilevel Cervical Epidural Hematoma After C5-6 Anterior Cervical Discectomy and Fusion: The Cry of a Neurosurgeon

**DOI:** 10.7759/cureus.39877

**Published:** 2023-06-02

**Authors:** Semih Can Cetintas, Rahsan Kemerdere, Taner Tanriverdi

**Affiliations:** 1 Neurosurgery, Istanbul University - Cerrahpasa, Cerrahpasa Medical Faculty, Istanbul, TUR; 2 Neurosurgery, Istanbul University - Cerrahpasa, School of Medicine, Istanbul, TUR

**Keywords:** post-neurosurgery complication, anterior cervical discectomy and fusion (acdf), cervical-spine, postoperative spinal epidural hematoma, cervical disc herniation

## Abstract

Anterior cervical discectomy and fusion (ACDF) is a safe and effective surgical treatment for cervical degenerative disk diseases. Almost every neurosurgeon is familiar with this approach. Anterior multilevel epidural hematoma (EDH) after a single ACDF is a very rare complication documented in the literature. There is no common consensus on the choice of optimal surgical treatment. Here, we report the case of a patient who showed multilevel EDH after ACDF at the C5-6 level to highlight that this complication should be kept in mind even after an uneventful surgery.

## Introduction

Anterior cervical discectomy and fusion (ACDF) is widely accepted as an effective and successful surgical procedure in degenerative cervical disc diseases, and almost every neurosurgeon has routinely performed this surgical approach. This approach may also result in several complications, including anterior and/or posterior epidural hematoma (EDH). It has been reported that the incidence of postoperative EDH after ACDF is very low, ranging up to 3%. Among the postoperative EDHs, the incidence of symptomatic EDH is even lower, ranging from 0.1% to 0.2% [1,2]. The majority of anterior EDHs are generally confined to the surgical site. However, anterior EDH far from the primary surgical site, called multilevel EDH, has been rarely described in a few cases [3,4]. Anterior EDH after ACDF, especially symptomatic ones, can be life-threatening, and prompt surgical intervention is the sine qua non. Because of the lack of enough data related to multilevel cervical anterior EDH in the current literature, a rapid decision and/or choice regarding the most appropriate surgical intervention may be challenging for a neurosurgeon.

Here, we describe the case of a patient who presented with multilevel anterior EDH after C5-6 ACDF and died on the seventh postoperative day. We want to describe “the cry of a neurosurgeon” to the young generation who, we hope, take some lesson(s) from our case.

## Case presentation

**Figure 1:**
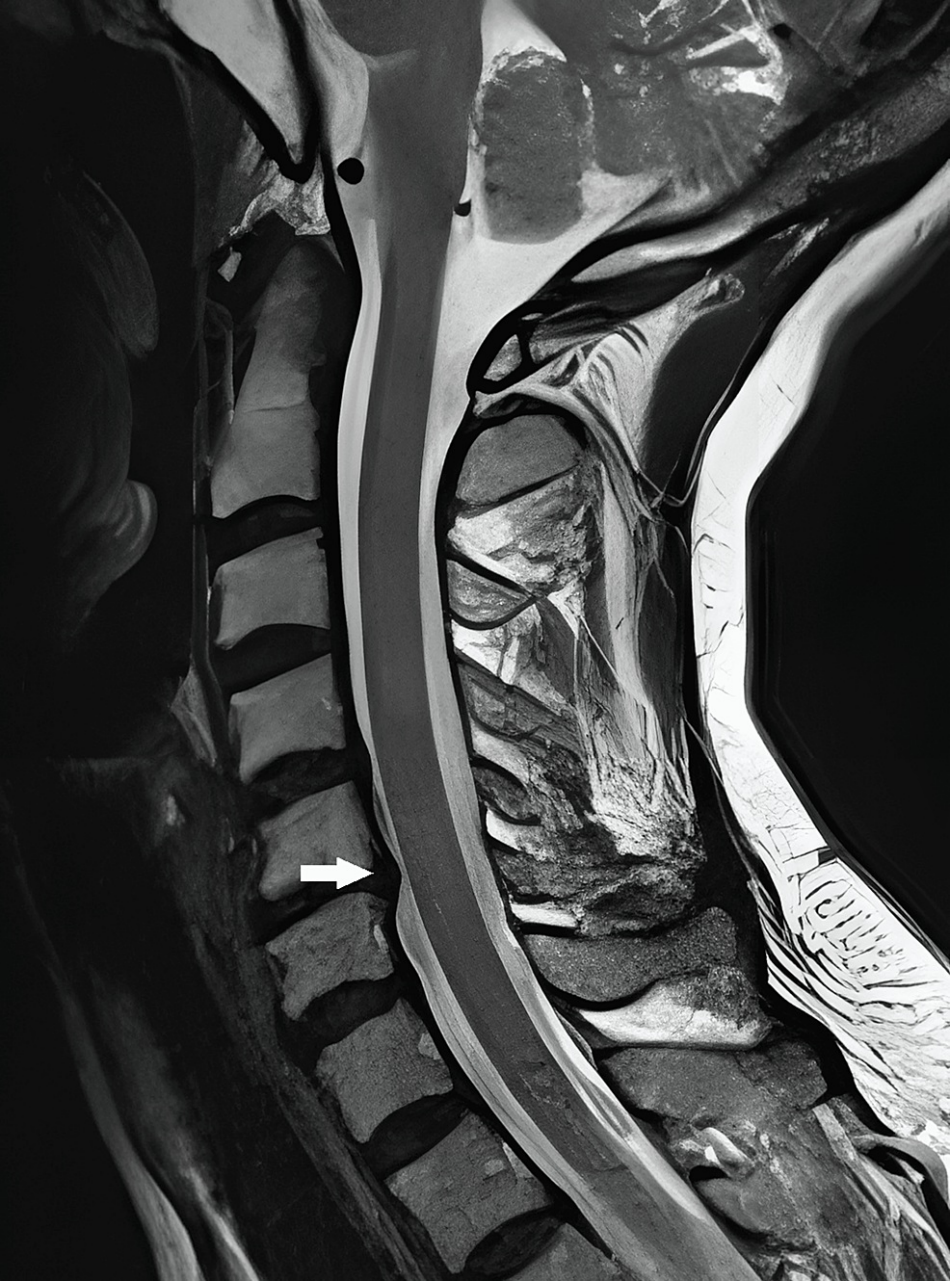
Sagittal T2-weighted MRI demonstrating a disc herniation at the C5-6 level (white arrow).

A 49-year-old female was admitted to our clinic for severe neck pain radiating to the right arm and fingers. Neurological examination was normal, and the patient reported taking levothyroxine 100 µg for hypothyroidism for years without any clinical disturbance. MRI of the cervical spine showed a disc herniation compressing the nerve root at the foraminal level on the right side at the C5-6 level (Figure 1). In the preoperative examination of the patient, thyroid hormone levels and platelet and coagulation parameters were found to be within normal values.

**Figure 2:**
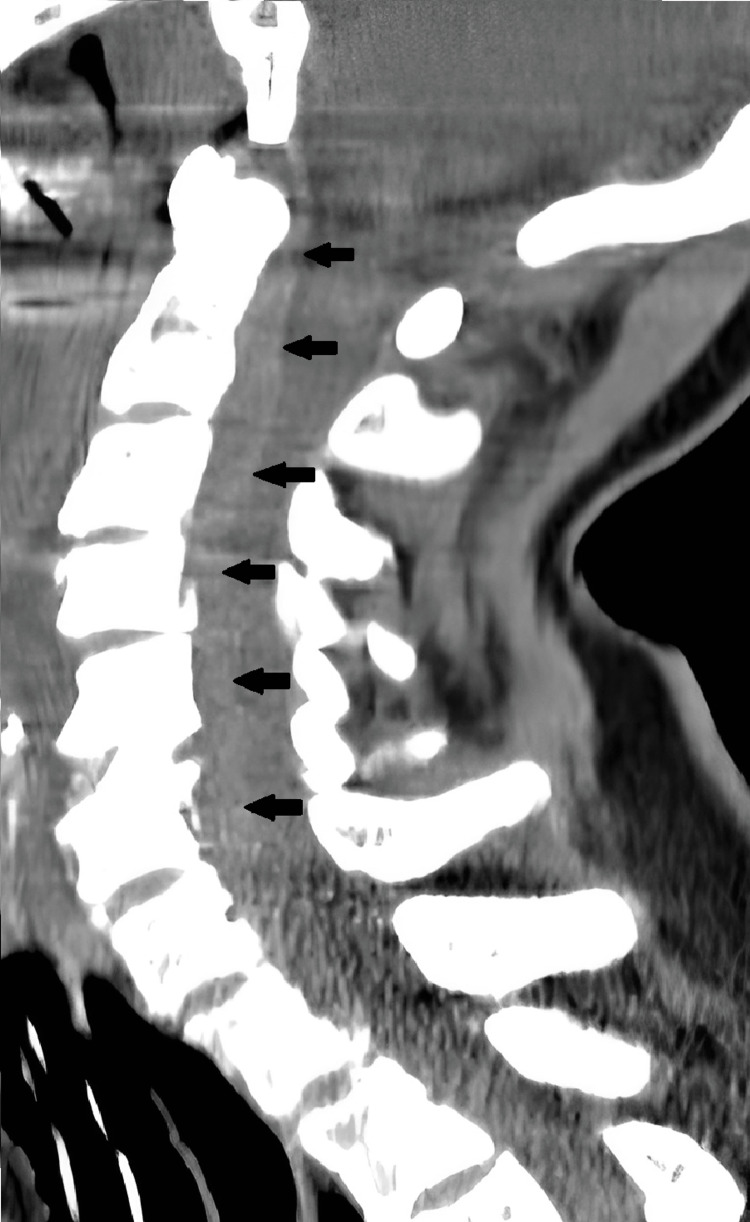
Cervical sagittal CT scan showing an anterior epidural hematoma extending from the clivus to the C6 (black arrows) and compression of the spinal cord.

The patient underwent ACDF and the decompression was completely achieved by opening the posterior longitudinal ligament. Following discectomy, a polyether-ether-ketone (PEEK) cage filled with an osteoinductive graft was positioned. The entire surgical procedure was uneventful and no bleeding was noted. As we perform usually, a submuscular drainage was placed before closing. Our anesthesiology team extubated the patient in the operating room and the patient was transferred to the recovery room. In the recovery room, early neurological examination was normal, following which the patient was transferred to our clinic. Four hours later, the patient became cyanotic and dyspneic without motor weakness. No swelling or bleeding was noted on neck inspection, and no bleeding was noted in the submuscular drain. Our anesthesiology team was called and the patient was intubated. Almost 10 minutes after intubation, we noted cardiac arrest and cardiopulmonary resuscitation was initiated. Almost seven minutes later, the heart rate returned to normal and the patient was transferred to the intensive care unit (ICU). Following the stabilization of the vital signs, a CT scan of the cervical spine was obtained, which showed an anterior EDH extending from the inferior end of the clivus to the inferior end of C6 (Figure 2).

**Figure 3:**
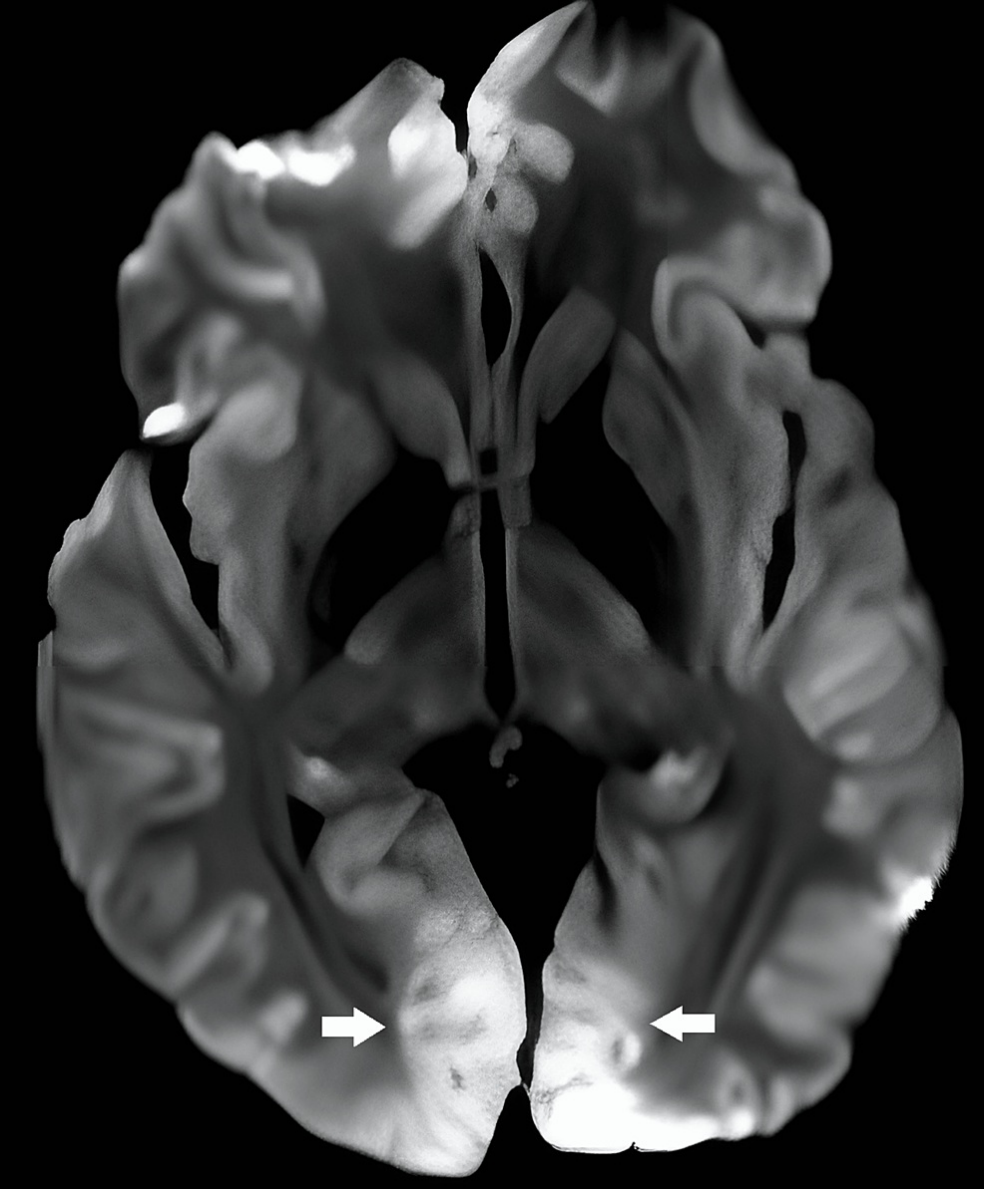
On the first postoperative day, brain diffusion MRI showed ischemia on both lateral occipital poles extending to the calcarine cortices (white arrows).

**Figure 4:**
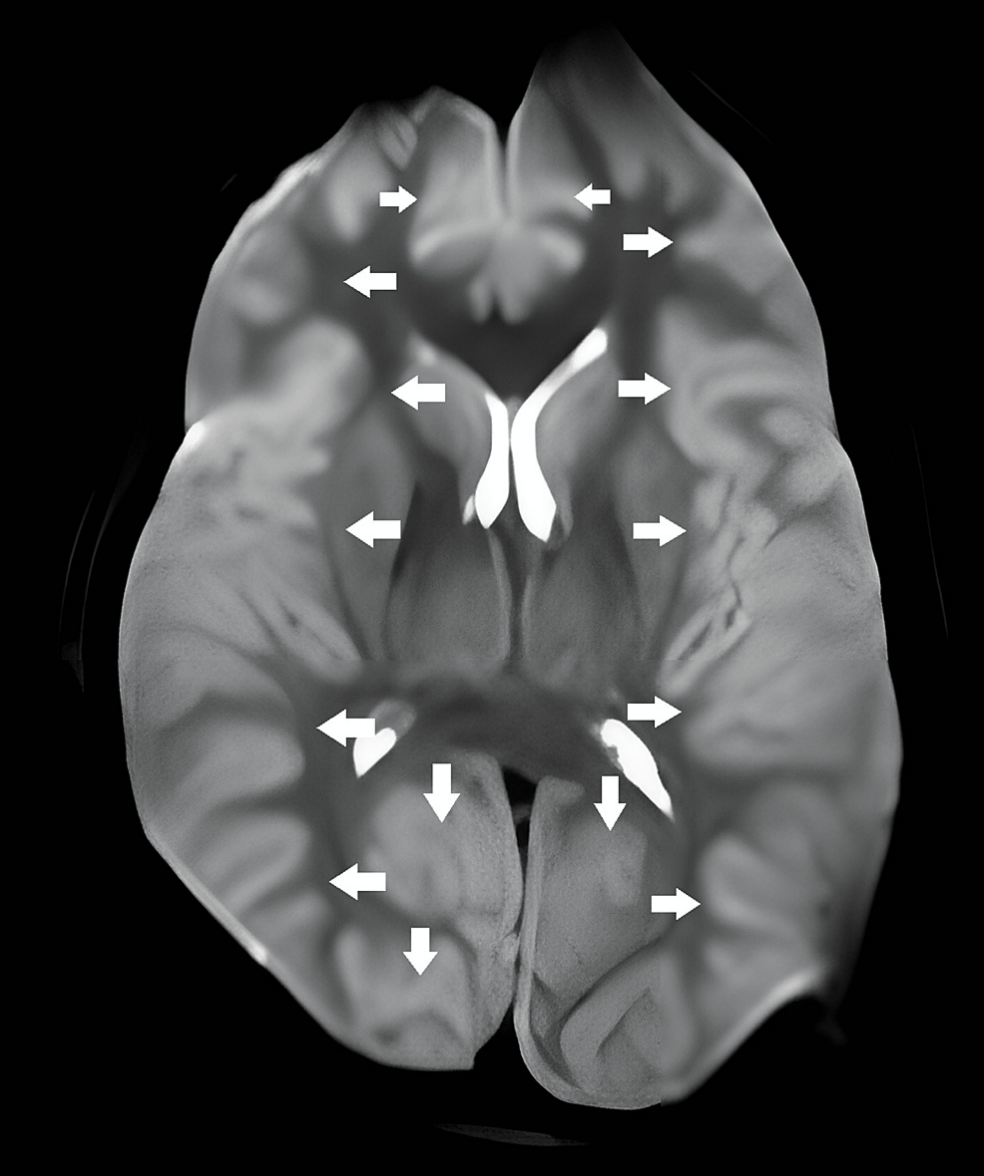
Diffuse hypoxic encephalopathy (white arrows) on brain diffusion MRI on the third postoperative day.

The patient was urgently transferred to the operating room for posterior decompression (almost one and a half hours after the initial onset of symptoms). During the surgery, laminectomy from C1 to C6 was performed. We noted that the hematoma was organized and it was not possible to evacuate. We performed multiple posterior laminectomies because we thought that a single-level anterior corpectomy would be insufficient to evacuate the hematoma. Thus, we aimed to provide adequate decompression even if we could not evacuate the hematoma. After enough decompression was achieved, two submuscular drains were placed posteriorly before closing. The second surgery was uneventful and the patient was transferred to the ICU without extubation. One day after the surgery, the patient started to have seizures and anti-epileptic medication was given. We thought that the seizure was due to possible brain ischemia because of cardiac arrest. Brain diffusion MRIs on day one and day three and cervical MRI on day one after surgery were obtained. Brain diffusion MRI (Figure 3) on day one showed ischemia on both lateral occipital poles extending to the calcarine cortices, and other areas of the brain appeared to be normal. On day three, diffusion MRI showed ischemic changes on the occipital poles had decreased, but new ischemic changes had appeared on upper cortices bilaterally (Figure 4).

**Figure 5:**
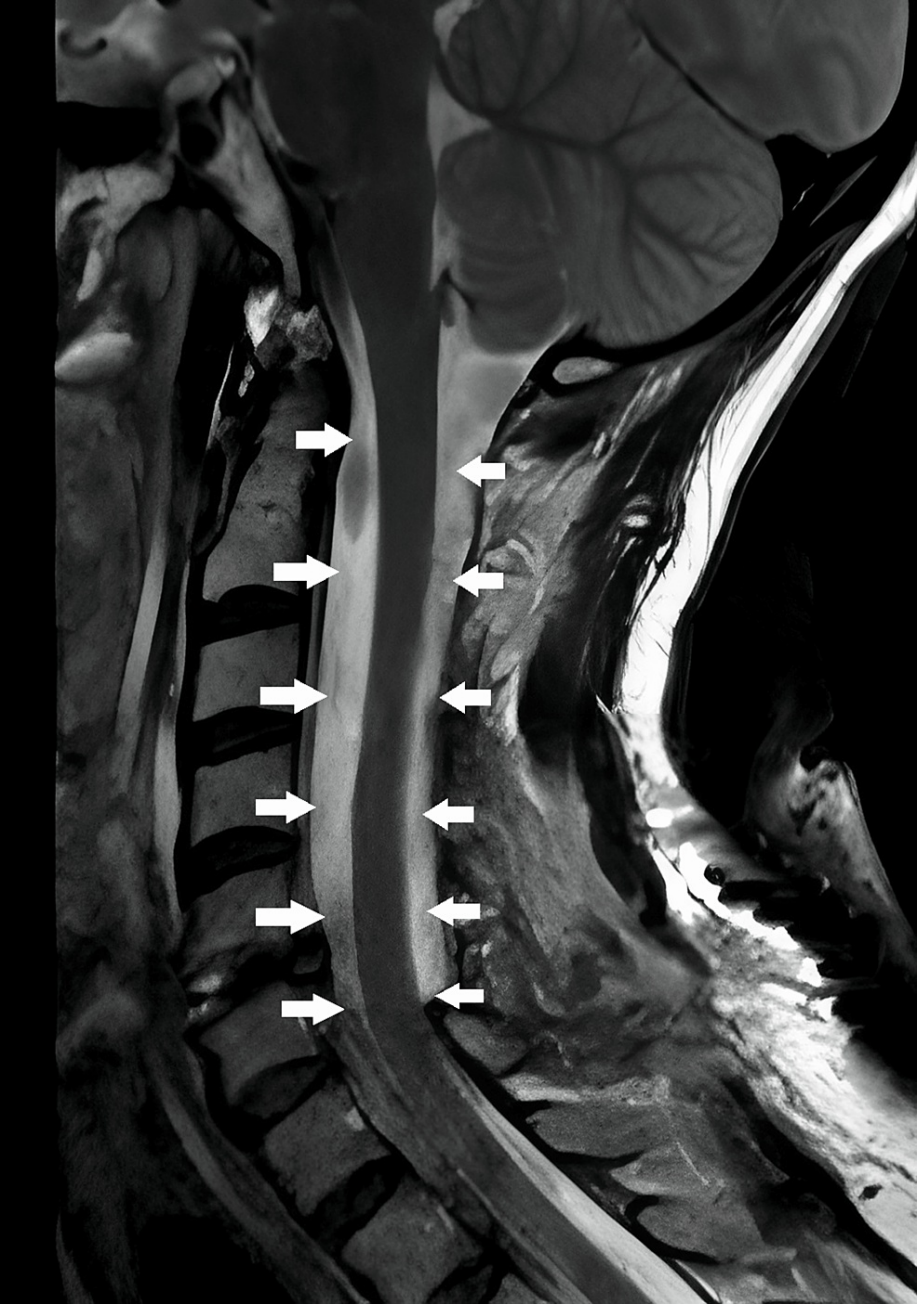
Sagittal T2-weighted MRI one day after the surgery showing no abnormality in the medulla and the achieved decompression (white arrows).

A cervical MRI (Figure 5) one day after the surgery showed that decompression was achieved, and there was no abnormal signal intensity in the cervical medulla. The patient could not be extubated during follow-up in the ICU unit, and, unfortunately, despite every effort, the patient died on the seventh day of the surgery.

## Discussion

Cervical anterior EDH is an uncommon complication related to ACDF and is generally confined to the primary surgical site. Anterior bleeding far from the surgical site or multilevel EDH in the cervical spine is very rare with a few cases reported in the literature [3-7]. Several risk factors are well-known by neurosurgeons, among which preoperative coagulopathies and multilevel surgical procedures are considered significant risk factors [8,9]. This complication may present with variable clinical disturbances but respiratory distress may be the first sign of anterior EDH [10]. In our case, there were no preoperative risk factors, and respiratory distress was the first sign.

What could be the explanation for the mechanism(s) of this unnoticed and/or unexpected complication? The limited data related to multilevel cervical anterior EDH in the current literature focuses on only one explanation, that is, injury to the anterior internal vertebral venous plexus located anterolaterally in the spinal canal [11]. It has been reported that this plexus can be injured during wide exposure such as the opening of the posterior longitudinal ligament or removal of osteophytes [3,4]. As these veins have no valve, a sudden increase in blood pressure because of coughing or Valsalva maneuver even during extubation can be directly transmitted to these veins, which start bleeding and can result in multilevel anterior EDH. In our patient, there was no obvious bleeding until the end of the surgery. Our anesthesiology team awakened the patient in the usual manner, and no hypertension was noted. One of the interesting radiological findings in our patient was that the thickest part of the EDH was found on the upper cervical spine although the patient’s head was in an elevated position at almost 45 degrees on the bed. We must agree with authors who experienced similar complications that wide decompression and placement of the PEEK cage resulted in hidden and large epidural space in which unnoticed or small injury to the plexus bled and filled the enlarged epidural space [3,4,7]. The bleeding did not stop because of the wide epidural space made by surgical decompression and went to the upper cervical levels. The literature underlines that unexpected EDH should be kept in mind, although the surgeon is confident of hemostasis and suggests that just before closing the case Valsalva maneuver may be performed for bleeding [3,4]. Our question is “how many neurosurgeons can consider this maneuver during ACDF?” We know that even serious venous bleeding during ACDF can be controlled by continuous saline irrigation and sometimes it even stops on its own.

When the anterior EDH is not multilevel or the hematoma is confined to the primary surgical site only, it is easy to choose the appropriate surgical intervention. The challenge is “what should be the appropriate surgical intervention in case of multilevel anterior EDH?” There is still a debate in the current literature about the choice of proper surgical intervention. The main reason is that very low incidence and lack of larger series prevent making a guide about the best management, but the common notion is that early detection and prompt surgical intervention lead to better outcomes [12]. In our case, we were as fast as possible to go to the operating room, which took one and a half hours from the onset of respiratory difficulty. If the neurological dysfunction shows rapid and progressive improvement, some supportive conservative treatments and close follow-up can be applied [3]. Some studies have suggested that revision of the previous surgical site should be the first choice [4,11,12]. Epidural placement of urinary [7] or lumbar catheter [4] for the removal of epidural blood by continuous saline irrigation has been reported. Other studies have advocated posterior decompression and blood evacuation [1]. In our patient, we performed posterior decompression, but, unfortunately, the blood could not be removed because it was organized. This situation leads to a question: “how can we remove the anterior coagulated or organized blood located far from the primary surgical site by irrigation through a catheter?” Thus, we are not sure that in cases in which prompt surgical intervention is required, it is difficult and time-consuming to adopt an anterior approach and posterior decompression could be the choice. Limited cases reported in the current literature showed us that the surgeons who dealt with these rare cases [1,3,4] were lucky because their patients did not have cardiac arrest as our patient did and did not deal with hypoxic encephalopathy as we did.

## Conclusions

Although the incidence is very low, multilevel anterior EDH following uneventful single-level simple cervical discectomy can be seen and leads to severe morbidity and even mortality. Depending on the limited data in the current literature and the present case, we cannot draw a conclusion related to the optimal treatment strategy. We highlight that prompt surgery, either anterior or posterior, should be performed depending on the choice of the surgeon. As a final word, a neurosurgeon’s cry is silent and there will always be tears in their eyes.
